# Impact of Gensingen brace treatment on Lenke 5 curvatures and chronic low back pain in late adolescent and adult scoliosis patients

**DOI:** 10.4102/sajp.v78i1.1585

**Published:** 2022-03-25

**Authors:** Budi S. Widjaja, Regina Varani

**Affiliations:** 1 Schroth Best Practice Academy, Neu-Bamberg, Germany; 2 Spine Clinic Family Holistic, Jakarta, Indonesia

**Keywords:** scoliosis, high correction bracing, Lenke 5C pattern, chronic back pain, loss of lumbar lordosis

## Abstract

**Background:**

Lenke 5C (lumbar and or thoracolumbar) curve patterns lead to loss of lumbar lordosis which is associated with low back pain in later adulthood. We undertook our study to investigate if brace treatment may have an effect on low back pain and on improving the cosmetic appearance in late adolescents and adults.

**Objectives:**

To estimate if conservative treatment may have an effect on pain in single lumbar curvatures in late adolescent and adult patients with Adolescent Idiopathic Scoliosis (AIS) using a Gensingen Brace by Weiss (GBW).

**Method:**

We investigated AIS patients with Lenke 5C pattern who wore a GBW prospectively. The inclusion criteria of our study were age over 15 years, Cobb angle greater than 20° before treatment and Risser 4 or 5. A verbal pain rating scale was used (no pain, mild pain, moderate pain, severe pain, very severe pain).

**Results:**

A total of 26 patients met the inclusion criteria. The average age was 17.7 years and the average Cobb angle was 41.5°. Nineteen patients (73.1%) experienced mild or moderate chronic low back pain before treatment and seven patients (26.9%) were asymptomatic but seeking treatment for cosmetic reasons. At follow-up, a 23% correction of the curve was achieved. All previously symptomatic patients reported that they no longer experienced low back pain after having worn the brace regularly.

**Conclusion:**

High correction bracing seems to have a positive effect on the curve and on chronic low back pain in patients with a scoliosis and a Lenke 5C curve pattern.

**Clinical implications:**

High correction, pattern specific bracing with a GBW may be applied aiming at reducing structural curves and chronic low back pain in late adolescent and adult patients with AIS and with a single lumbar curvature.

## Introduction

Scoliosis is a three-dimensional deformity of the spine and the trunk and may lead to functional impairment and pain (Asher & Burton 2006; Goldberg et al. 2002; Weinstein et al. 2003). Malformations or synostosis of vertebrae and ribs, neuromuscular diseases or mesenchymal disorders besides other rare conditions may coincide with a spinal curvature. However, the most common form of all scoliosis conditions is called ‘idiopathic’, which comprises 85% of all cases. The underlying cause of idiopathic scoliosis is not yet fully understood (Asher & Burton 2006; Kenner et al. 2019; Kruzel & Moramarco 2020). A functional tethering of the spinal cord as found by Deng et al. (2015) as a reason for the ventral overgrowth within the thoracic spine (Chu et al. 2006) currently seems the most promising concept explaining the aetiology of adolescent idiopathic scoliosis (AIS). This concept was the basis for a new functional treatment approach, including so-called de-tethering exercises and a special technique of extracorporal shockwave therapy (ECSWT; Weiss 2017).

Late-onset idiopathic scoliosis or AIS in principle has a benign prognosis and – even when untreated – rarely leads to serious health conditions (e.g. cardiopulmonary impairment or impairment of the nervous system) other than low back pain and cosmetic concerns (Weinstein et al. 2003; Weiss, Moramarco & Moramarco 2016). However, AIS is not a uniform condition. It appears with different curve patterns which may be named according to the location of the main curve like thoracic, lumbar, double major or thoracolumbar curves (Asher & Burton 2006; Kruzel & Moramarco 2020). Currently, for pattern-specific physiotherapy and for bracing, the Rigo- or the Augmented Lehnert-Schroth (ALS)-classification is used, (Rigo, Villagrasa & Gallo 2010; Weiss 2010) and for surgery the Lenke classification has been established (Lenke et al. 1998). The ALS classification is widely used and consists of seven patterns of curvature. Historically, the patterns within the Lehnert-Schroth (LS) classification are subdivided into 3-curve (3C) and 4-curve (4C) patterns (Borysov et al. 2020). While in the 3C patterns the thoracic curve is structural and dominant, in the 4C patterns it is the structural lumbar curve which takes the lead. The ALS-classification provides a further subclassification of 3C and 4C patterns according to the length of the major curve and of the existing nonstructural counter curves (Chik 2020; Rothstock et al. 2020; Weiss 2010).

Curve progression may appear fast during phases of rapid growth of a child or in early adolescence, because the greater the rate of growth, the greater the risk of an increase in curvature (Asher & Burton 2006; Goldberg et al. 2002; Kruzel & Moramarco 2020). At the end of growth or in adulthood, only curves above 30° – 40° Cobb angle tend to progress slowly over the decades (Asher & Burton 2006; Kruzel & Moramarco 2020). In a frontal plane X-ray the Cobb angle is measured and the maturity can be determined from the ossification of the iliac apophysis. According to Risser, there are six stages of skeletal maturity. Risser 0 (apophysis not visible) is the stage usually before the onset of menarche in girls or voice change in boys. After menarche and voice change Risser 1 to 5 appear with Risser 4 being about 99% outgrown and Risser 5 outgrown in full (Risser 1958).

Many AIS patients come for treatment at 12.8 ± 2.1 years old, with mean Cobb angles of 47.88° ± 14.28° at their first visit. The asymmetry is frequently noticed mostly by their mothers, when patients are in the shower, swimwear or wearing form-fitting clothes (Kenner et al. 2019). From our clinical experience we assume that in Indonesia, Muslims usually wear loose-fitting attire, and this might increase the likelihood of late detection of scoliosis. Moreover, Lenke 5 patterns do not affect the ribcage, thus the deformity is less obvious. Lenke 5 patterns (single lumbar / thoracolumbar curves) lead to loss of lumbar lordosis which is associated with low back pain in later adulthood (Asher & Burton 2006; Djurasovic & Glassman 2007; Glassman et al. 2005). It has already been shown that restoring lumbar lordosis with a specific brace may reduce chronic low back pain in adults with a scoliosis (Weiss & Turnbull 2019). The purpose of our study was to investigate if brace treatment has the potential to change the natural history of Lenke 5 curvatures in late adolescents and in adults (maintaining and or correcting the curves), and to test if this approach may also have an influence on low back pain in this population.

## Method

We investigated patients with AIS and with a Lenke 5C pattern who wore a Gensingen brace (GBW) which is produced based on a computer-aided design aiming at a three-dimensional (3D) correction of the spine and trunk and at a restoration of the sagittal profile (Weiss et al. 2019). The brace adjustment and management were conducted in our clinic within our usual routine by both authors.

**FIGURE 1:**
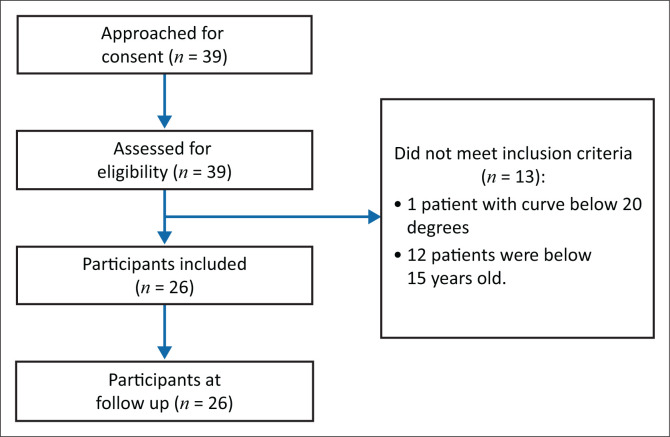
Recruitment flowchart of study participants.

A retrospective chart review of a cohort gathered prospectively was undertaken including all patients with a Lenke 5C pattern who were treated between January 2018 and June 2019 with the following inclusion criteria: age > 15 years but before menopause, Lenke 5C pattern scoliosis, Cobb angle > 20° and Risser sign 4 or 5. The exclusion criteria were as follows: patients with scoliosis of other origins than AIS and those with a history of spinal surgery. Therefore, only patients without the immediate risk of a fast progression and without the typical signs of degeneration during or after menopause were included (Figure 1).

### Outcome measures

The patients were assessed at the beginning by (1) measuring the initial trunk rotation using a Scoliometer™, (2) the severity of the Cobb angle, and (3) asking the patients if they had any pain symptoms related to the scoliosis. A verbal pain rating scale (VRS) with a 5-point scale was used (no pain, mild pain, moderate pain, severe pain, very severe pain) to measure the degree of back pain they had experienced before wearing a brace.

After the brace was fitted to the patients, they had an X-ray examination while in the brace, and the exact fit of the brace as well as the correction of the curve as obtained was measured. These in-brace corrections (Cobb angle without brace – Cobb angle in brace = In-brace correction) were documented and the patients were followed up after 6 months while wearing the brace. All the outcome parameters were reassessed at follow-up.

### Procedures

The Cobb angle measurements were performed by one of the authors, who is an experienced medical physician with more than 5 years of experience within the field and the X-rays were taken in the radiological department outside our clinic. They were not blinded for the measurements.

The patients were also given six sessions of Schroth Best Practice (SBP) exercises by one of the authors (Weiss, Moramarco & Borysov 2020) at the beginning of treatment, and were instructed to exercise regularly with a minimum of twice a week. However, the exercise compliance was not measured in our study.

Patients with good in-brace correction have been shown to have a better result (Yrjonen 2007). Thus, in our practice, above average in-brace correction was defined as a reduction of the Cobb angle of > 50% on X-ray. This limit was set in accordance with the findings in the last GBW studies (Weiss et al. 2019, 2021). Average corrections of around 50% in the GBW were documented there.

At the 6 months follow-up, the patients underwent another X-ray investigation and the VRS was used again. The bracing method was considered effective when the curve was improved by 5% or more, and significant correction was defined as a Cobb angle reduction of > 20%.

The patients were asked if they followed the scheduled wearing time of the brace at follow-up. The scheduled wearing time was a minimum of 20 h. All patients involved were following the regimen, but exact brace wearing time was not specified. Gender difference was not considered.

### Data analysis

Data analysis was performed with Statistical Package for the Social Sciences (SPSS) version16 for Windows (IBM, Armonk, NY, USA.) Normality analysis was performed with a Shapiro–Wilk test. Descriptive data were reported as frequencies and percentages or means and standard deviations (s.d.) as appropriate. Pre-treatment and post-treatment measurements were compared using the Wilcoxon signed-rank test as the data were not normally distributed (*p* < 0.05) and thus this non-parametric statistical test was used to analyse the data.

The indication for treatment was made independently of inclusion in our study and only these measures were used (X-ray, Angle of Trunk Rotation [ATR] measurement, physiotherapy, brace fitting) which are generally recognised, and evidence-based. Consent had been obtained from the patients and in the case of minors from their legal guardians for publication of their pictures, provided that their names were not mentioned. No personal data were passed on that could have led to the identification of the included patients. All patients who can be seen in the pictures had given their consent for the publication of their pictures in writing.

### Ethical considerations

This article followed all ethical standards for research without direct contact with human or animal subjects.

## Results

There were a total of 39 patients with Lenke 5C curve patterns treated at the clinic between January 2018 and June 2019. Thirty-nine patients were assessed for eligibility and only 26 met our inclusion criteria and were all included in our study (Figure 1). The mean age was 17.6 years (17.6 ± 5.2) and the majority (73.1%) were female. The majority were symptomatic for pain and there were 26.9% who were asymptomatic, but sought treatment for cosmetic purposes (Table 1).

**TABLE 1:** Descriptive characteristics of the patients (*n* = 26).

Characteristics	Description			
	Mean (s.d.)	Range	*n*	%
**Age (years)**	17.6 ± 5.2	15–40	-	-
**Height (cm)**	161 ± 7.1	147–175	-	-
**Risser**	4.4 ± 0.6	3–5	-	-
**Gender**				
**Female**	-	-	19	73.1
**Male**	-	-	7	26.9
**Presence of back pain**				
**None**	-	-	7	26.9
**Mild**	-	-	11	42.3
**Moderate**	-	-	8	13.0
**Severe or very severe**	-	-	0	0
**Verbal rating scale**	2.03 ± 0.7	-	-	-

s.d., standard deviation.

Mean Cobb angle value obtained before the treatment was 41.5° ± 13.4° (Table 2) and mean Cobb angle value measured in-brace was 17.1° ± 15.5°. In-brace correction was 67% (average in-brace Cobb angle 17°). After follow-up at 6 months, correction of 23% was achieved (average Cobb angle 33.2°). We observed that four patients (15.4%) showed less than 5% reduction in Cobb angle, but 22 patients (84.6%) showed an improvement of 5% or more. From this group, 12 patients (54.5%) experienced a significant correction of 20% or more. A statistically significant difference was obtained when Cobb angles before the treatment and in-brace were compared (*p* < 0.001), and when compared to Cobb angles at 6 months follow-up (*p* < 0.001).

**TABLE 2:** Comparison of measurements obtained before and 6 months after treatment.

Variable	Pre-treatment	In brace	Follow up (6 months)
Cobbs, Mean (s.d.) in degrees	41.5 ± 13.4	17.1 ± 15.5	33.2 ± 15.2
% correction	Not applicable	67%	23%
Angle of Trunk Rotation (ATR), Mean (s.d.) in degrees	12.8 ± 4.8	Not applicable	6.4 ± 4.5
Verbal rating scale, mean (s.d.)	2.03 ± 0.7	Not applicable	0

s.d., standard deviation.

**FIGURE 2:**
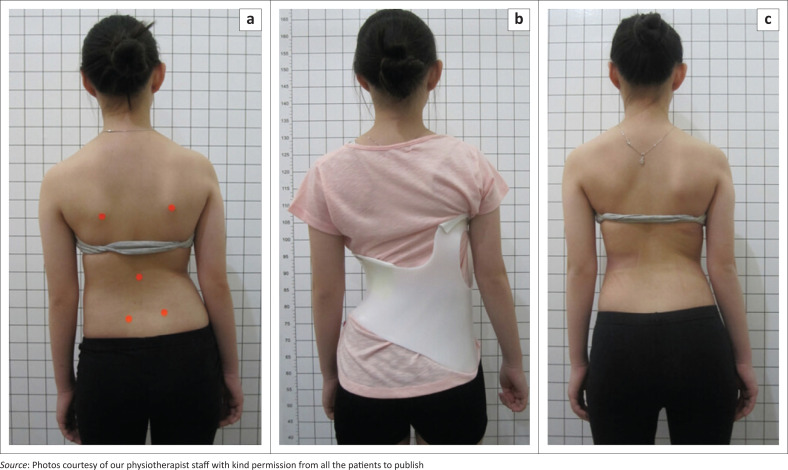
Cosmetic improvement in a patient with a left thoracolumbar curve (a) as treated with a Gensingen brace (b) for this pattern showing a reasonable cosmetic improvement after the follow-up of 6 months (c). Cobb angle has been reduced.

**FIGURE 3:**
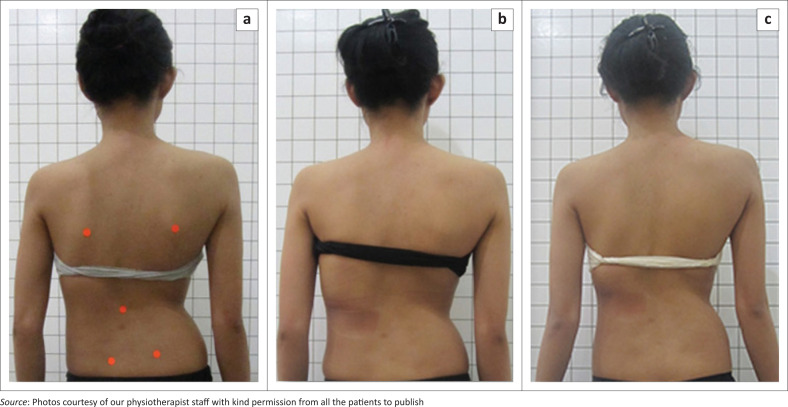
Twenty-three-year-old patient with a left thoracolumbar curve initially of 52° (a) with a marked improvement of her physical appearance after the follow-up period (b, c).

Nineteen patients (73.1%) experienced chronic low back pain of mild to moderate degree before the treatment and seven patients (26.9%) were asymptomatic, seeking treatment for cosmetic reasons. At 6-months follow-up, all previously symptomatic patients reported that they no longer experienced low back pain. Thus, bracing may be considered to have the potential to be effective with respect to Cobb angle reduction and pain control (Figure 2 and Figure 3).

## Discussion

The aims of treatment in our patients were (1) a reduction of scoliosis curvature and (2) reduction of pain. From the results we can see that there is a mean decrease in Cobb angle severity about 23% and ATR measurement from 12.8° ± 4.8° to 6.4° ± 4.5° at 6 months follow up, and the pain symptoms were reportedly absent.

Bracing is considered beneficial during growth when the rate of progression was reduced or when the angle of curvature was improved, and this improved the cosmetic appearance as well (Weinstein 2013, Weiss et al. 2019, 2021). Usually after growth, the application of high correction braces is rarely used because there is limited evidence of an impact of these braces on deformity and pain in patients with scoliosis (Weiss & Turnbull 2019).

Scoliosis affects the sagittal profile and usually leads to thoracic flattening and lumbar kyphosis. Especially, in Lenke 5 patterns usually a lumbar or thoracolumbar kyphosis is evident. Therefore, braces for the correction of a lumbar kyphosis and restoring a lumbar lordosis have been applied successfully. With minor curves a sagittal realignment brace (physio-logic brace™)has been in use addressing the sagittal plane only (Weiss & Werkmann 2009a, 2009b). In scoliosis with greater 3D spinal deviations, Chêneau style braces (Rigo brace or GBW) are applied as these also improve the sagittal plane deformity which is usually found in patients with idiopathic scoliosis (loss of thoracic kyphosis, loss of lumbar lordosis, flat back deformity (Weiss et al. 2016).

In a prospective study it has been shown that physiotherapy may have a beneficial effect on the pain levels in patients with scoliosis and pain (Zapata et al. 2015). However, in a certain proportion of patients the application of physiotherapy alone is insufficient. For these cases, specific bracing has also been proposed (Weiss et al. 2016). While unspecific braces have no clear effect on pain levels (Alaranta & Hurri 1988; Jellema et al. 2001, 2002; Van Poppel et al. 1998), braces specifically addressing the sagittal plane deformity have been shown to be effective (Weiss & Werkmann 2009a, 2009b).

In a certain proportion of patients with scoliosis and pain, the restoration of a lumbar lordosis may not be helpful or may even be contraindicated, because increasing lumbar lordosis in these patients might also increase the pain (Weiss & Werkmann 2009a). These are patients with functional or structural (spondylolisthesis) instabilities (Weiss & Werkmann 2009a). A simple functional test to classify the type of low back pain has been proposed (Weiss & Werkmann 2009a, 2009b). For instability low back pain patients, symmetric braces are in use for reducing a lumbar lordosis (Weiss & Werkmann 2009a). The GBW can also be designed in this way to help stabilize the lumbar segment should a 3D correction be necessary.

From our study, we can see that during the 6 months period of bracing with a 3D high corrective brace, there was still achievable correction on X-ray (23%) even in late adolescent or adult patients, even though the value is of varying degree. It is also still inconclusive, yet the treatment is still ongoing given the brace treatment will continue for 2 years of wearing time to achieve more stability in the correction. But aside from that, the cosmetic appearance did improve as can be seen in our before and after clinical pictures (Figure 2 and Figure 3).

This is also confirmed with the improvement in trunk rotation as measured in ATR and the improvement in pain symptoms. Thus, our study can be viewed as a pilot study showing that pain and deformity may be reduced when applying high correction braces. The primary aim for the patients in our study was to reduce the deformity, pain being a secondary issue.

## Limitations

Our study was designed as a prospective short-term follow-up without an untreated control group. The sample size was small. Therefore, this investigation may be viewed as a pilot investigation while there are limited reports on patients out of the growth phase treated with a brace (Weiss & Turnbull 2019). Cosmetic improvements have been documented for adolescents with a scoliosis in the growth phase while it is still under debate whether there may be a cosmetic benefit of bracing at the end or after growth is completed (Weiss et al. 2019, 2021). Therefore, despite the small sample size, our study adds to the limited body of evidence on the application of braces in patients with spinal deformities at or after the end of growth.

## Conclusion

High correction specific bracing with GBW seems to be effective in maintaining or reducing the curvature in late adolescent and adult AIS patients with single lumbar or thoracolumbar curvatures. Additionally, the GBW application seems to have an influence on chronic pain in patients with a lumbar or thoracolumbar curvature.

However, because of the small sample size and the study design, a conclusion cannot be drawn.

Nevertheless, the findings from our study seem promising enough to justify undertaking a prospective controlled or randomised controlled study to gain definite evidence for or against brace treatment in mature individuals with scoliosis and with cosmetic concerns and or chronic low back pain.
